# Development of Subcutaneous Panniculitis-Like T-cell Lymphoma After Immune Checkpoint Inhibitor Treatment

**DOI:** 10.7759/cureus.66974

**Published:** 2024-08-15

**Authors:** Tommy Y Yu, Zartash Gul, Alicia M Hunt, Michael J Williams, Alexander M Maley

**Affiliations:** 1 Department of Medicine, University of Wisconsin School of Medicine and Public Health, Madison, USA; 2 Department of Oncology, Aurora St. Luke's Medical Center, Milwaukee, USA; 3 Department of Pathology and Laboratory Medicine, Aurora St. Luke's Medical Center, Milwaukee, USA; 4 Department of Pharmacy, Aurora St. Luke's Medical Center, Milwaukee, USA; 5 Department of Dermatology, Aurora St. Luke's Medical Center, Milwaukee, USA

**Keywords:** immune checkpoint inhibitor, cutaneous t-cell lymphoma, nivolumab-related adverse events, cancer immunotherapy, melanoma treatment

## Abstract

Program death 1 (PD-1) inhibitors such as nivolumab are immune checkpoint inhibitors that have revolutionized the treatment of metastatic melanoma. Despite its success in treating melanoma, immune activation can lead to immune-related adverse effects, which are experienced by half of melanoma patients treated with PD-1 inhibitors. Despite the common frequency of immune-mediated adverse events, the development of a secondary lymphoma is exceedingly rare. We present the case of a 53-year-old woman diagnosed with stage IV metastatic melanoma, treated with nivolumab, who subsequently developed fatal subcutaneous panniculitis-like T-cell lymphoma (SPTCL).

## Introduction

Immune checkpoint inhibitors are targeted monoclonal antibodies which block T-cell inactivation receptors which are used by malignant cells to evade the body's immune system. Through this mechanism, program death 1 (PD-1) inhibitors result in T-cell activation and proliferation. Subcutaneous panniculitis-like T-cell lymphoma (SPTCL) is a rare primary cutaneous lymphoma derived from cytotoxic alpha-beta T cells. Most cases of SPTCL are indolent with a good prognosis. However, if complicated by hemophagocytic lymphohistiocytosis (HLH), the prognosis is poor. Nivolumab is a PD-1 inhibitor that has become the standard of care in the treatment of metastatic melanoma. Here, we present a rare case of a 53-year-old woman with stage IV metastatic melanoma, treated with nivolumab, who later developed SPTCL.

## Case presentation

A 53-year-old woman presented with stage IV metastatic melanoma of the mid-upper back. She was treated with four cycles of nivolumab and ipilimumab followed by 12 cycles of nivolumab alone. She had a complete response based on PET-CT imaging. 

**Figure 1:**
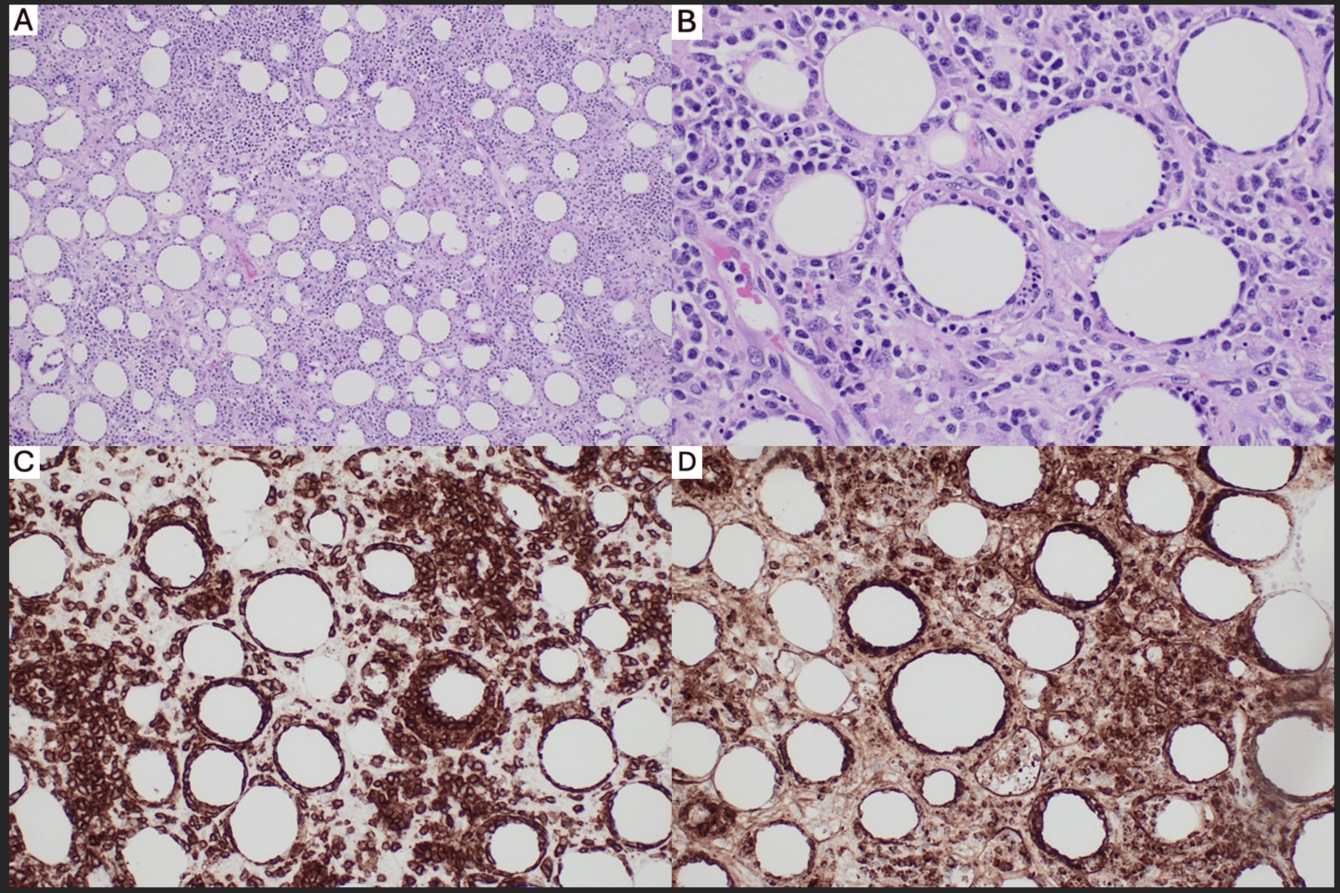
Hematoxylin and eosin stain at 10× (A) and 40× (B) showing subcutaneous infiltrate of atypical lymphocytes rimming the adipocytes, which are positive for CD8 (C) and granzyme B (D), consistent with subcutaneous panniculitis-like T-cell lymphoma.

Two months after her last nivolumab infusion, she developed numerous indurated subcutaneous nodules on the trunk and bilateral upper extremities along with systemic complaints of fatigue, fevers, night sweats, and rigors. Excisional biopsies were performed from the abdomen and shoulder. The histopathological exam showed a lymphocytic infiltrate predominately present around the subcutaneous adipocytes and rimming the adipocyte membrane (Figure 1). Immunohistochemical staining was positive for CD3, CD8, granzyme B, and the alpha-beta T-cell receptor. Immunohistochemistry was negative for CD56, CD30, and Epstein-Barr virus (EBV).

The patient was diagnosed with SPTCL. Treatment was begun with combination chemotherapy of cyclophosphamide, doxorubicin, etoposide, vincristine, and prednisone. She received four cycles of chemotherapy. Three months after her diagnosis of SPTCL, her disease was complicated by HLH with rapid progression. She was febrile with the following relevant laboratory values: ferritin 10,897 ng/mL, C-reactive protein (CRP) 8.7 mg/dL, triglycerides 175 mg/dL, red blood cell count 6.7 g/dL, platelets 18 k/mcl, and soluble IL-2 receptor 2387 pg/mL. CT scan of the abdomen showed splenomegaly; the spleen measured 16.2 cm in craniocaudal dimension. She was admitted to the intensive care unit, where she was diagnosed with septic shock complicated by respiratory and renal failure and was placed on a ventilator. She was treated with a wide spectrum of antimicrobials, including vancomycin, cefepime, acyclovir, and fluconazole, as well as multiple vasopressors such as phenylephrine, epinephrine, norepinephrine, and vasopressin. Despite these interventions, she remained hypotensive and persistently hypoxemic. Bronchoscopy revealed a cavitary lesion and necrotic lung tissue. Culture from bronchoalveolar lavage cytology grew *Rhizopus microsporus*. She subsequently passed away after a three-day admission. 

## Discussion

PD-1 is a key checkpoint molecule that, upon binding with one of its ligands PD-L1 or PD-L2, exhibits inhibitory function in T cells, including the prevention of anti-tumor immunity. By blocking this function, PD-1 inhibitors such as nivolumab trigger an anti-tumor immune response that has been transformative in the treatment of multiple types of cancer.

While the use of PD-1 inhibitors is commonly associated with immune-related adverse events, the development and progression of T-cell lymphomas are exceedingly rare. A phase II study using nivolumab for the treatment of refractory peripheral T-cell lymphoma was terminated due to the hyperprogression of lymphoma in four of 12 patients [1]. In another phase II trial in patients with adult T-cell leukemia-lymphoma (ATLL), all three patients developed rapid progression of their lymphoma after a single dose of nivolumab [2]. In addition to leading to the hyperprogression of T-cell lymphoma, PD-1 inhibitors have also been reported to lead to the de novo development of T-cell lymphoma in multiple case reports [3,4].

The exact underlying mechanism of this rare and seemingly paradoxical complication is unknown. In a murine T-cell lymphoma model, anti-PD-1 antibodies induced massive lethal lymphoproliferation, suggesting that PD-1 not only acts as an immune checkpoint but also functions as a tumor suppressor for T cells [5]. In the terminated trial using nivolumab to treat ATLL, the authors analyzed clonality, somatic mutations, and gene expression in the malignant cells to confirm rapid clonal expansion after PD-1 blockade [6]. Their proposed mechanisms for the hyperprogression of ATLL after nivolumab include PD-1 blockade driving the expression of additional checkpoints that promote the suppressive activity of ATLL cells, PD-1 blockade altering the expression of ATLL-promoting growth factors, stromal PD-L1 acting as a tumor suppressor by binding to PD-1 expressed on leukemic cells in the ATLL microenvironment, and PD-1 uniquely governing immunity in patients with chronic or smoldering ATLL. To our knowledge, this is the first case documenting the development of SPTCL following treatment with PD-1 inhibitor nivolumab. In a pooled report of four melanoma trials of anti-PD-1 monotherapy, 49% of patients experienced immune-related adverse events, but there were no cases of lymphoma [7]. SPTCL is a rare primary cutaneous lymphoma composed of alpha-beta cytotoxic T cells [8]. Histologically, the neoplastic T cells express CD8, beta F1, and other cytotoxic T-cell markers such as granzyme B, perforin, and TIA1 [8]. The neoplastic T-cell infiltrates tend to surround the rim of adipocytes and distort their membrane. While most cases of SPTCL have a rather indolent course and good prognosis, our patient's disease was further complicated by HLH, a syndrome of macrophage dysfunction that can result in critical illness [8]. HLH was diagnosed by her combination of fever, anemia, thrombocytopenia, hypertriglyceridemia, elevated ferritin, soluble IL-2 receptor, and splenomegaly. HLH is seen in up to 20% of patients diagnosed with SPTCL and is associated with a poor prognosis, with a five-year survival rate of 46% compared to 91% of those without this complication [8]. There is currently no standard treatment regimen for SPTCL. Unfortunately, our patient progressed despite early treatment with chemotherapy, succumbing to shock complicated by an invasive pulmonary fungal infection.

## Conclusions

Nivolumab and other PD-1 inhibitors play a central role in melanoma treatment. Progression of primary T-cell lymphomas and development of secondary T-cell lymphomas are possible immune-related adverse events following PD-1 inhibitor treatment. Physicians should be aware of this rare and potentially fatal adverse immune event. 
